# Effects of insulin-like growth factor-induced Wharton jelly mesenchymal stem cells toward chondrogenesis in an osteoarthritis model

**DOI:** 10.22038/IJBMS.2018.28205.6840

**Published:** 2018-07

**Authors:** Wahyu Widowati, Ervi Afifah, Tjandrawati Mozef, Ferry Sandra, Rizal Rizal, Annisa Amalia, Yukko Arinta, Indra Bachtiar, Harry Murti

**Affiliations:** 1 Medical Research Center, Faculty of Medicine, Maranatha Christian University, Bandung 40164, West Java, Indonesia; 2 Biomolecular and Biomedical Research Center, Aretha Medika Utama, Bandung 40163, West Java, Indonesia; 3 Research Center for Chemistry, Indonesian Institute of Sciences, Serpong, Indonesia; 4 Department of Biochemistry and Molecular Biology, Faculty of Dentistry, Trisakti University, Jakarta, Indonesia; 5 Stem Cell and Cancer Institute, Jakarta 13210, Indonesia

**Keywords:** Chondrogenesis, IGF-1, Mesenchymal stem cell MMPs, Osteoarthritis

## Abstract

**Objective(s):**

This study aimed to determine the collagen type II (COL2) and SOX9 expression in interleukin growth factor (IGF-1)-induced Wharton’s Jelly mesenchymal stem cells (WJMSCs) and the level of chondrogenic markers in co-culture IGF1-WJMSCs and IL1β-CHON002 as osteoarthritis (OA) cells model.

**Materials and Methods:**

WJMSCs were induced with IGF1 (75, 150, and 300 ng/ml) to enhance their chondrogenesis capability. The gene expression of SOX9 and COL2 was evaluated with quantitative RT-PCR. Furthermore, IGF1-WJMSCs were co-cultured with IL1β-CHON002 cells in varied ratios (1:2, 1:1, 2:1). Chondrogenic markers ADAMTS1, ADAMTS5, MMP3, MMP1, and RANKL were measured with ELISA.

**Results:**

The IGF1-WJMSCs had an increased expression of COL2 and SOX9. ADAMTS1, ADAMTS5, MMP1, MMP3, and RANKL levels were decreased in the co-culture IGF1-WJMSCs and IL1β-CHON002.

**Conclusion:**

The IGF1-induced WJMSCs were capable to enhance chondrogenesis, indicated by increased expression of SOX9 and COL2 and decreased expression of ADAMTS1, ADAMTS5, MMP3, MMP1, and RANKL. These findings can be further used in the osteoarthritis treatment.

## Introduction

Osteoarthritis (OA) is one of the common joint disorders worldwide and the main cause of body-support disability. The characteristics of OA include phenotypic changes in the superficial layer cells of the articular cartilage (AC), chondrocyte hypertrophy and apoptosis, progressive formation of osteophyte, fibrillation of the AC, sclerosis of subchondral bone, and increased remodeling of the periarticular bone (1–3). 

The chondrocyte phenotype is characterized by specific genes expressions, i.e., collagen type II and the transcription factor SOX9 (4, 5). COL2 is an essential abundant component in the cartilage extra cellular matrix (ECM). Therefore, the COL2 disruption and loss of other cartilage ECM components during degenerative joint diseases such as OA will lead to severe disability and aging-related health problems (6). This may be stimulated by complex pathogenic mechanisms that decreased matrix synthesis and upregulated pathways of tissue degradation (7). 

Stem cells take a role in novel treatment strategies for both clinical situations. Johnson *et al.* discovered a drug candidate by screening the small molecules that induced mesenchymal stem cells (MSCs) chondrogenic differentiation (8). MSCs were studied for cartilage development (9), which may be helpful for the developmental programs in OA.

MSCs were collected from cartilage (bone marrow mesenchymal stem cells/BM-MSCs) and subsequently from adipose tissue, the placenta, dental pulp, umbilical cord, amnion (10,), and Wharton’s Jelly (11). Adipose tissue-MSCs (AD-MSCs) have lower chondrogenesis ability than BM-MSCs. Induction of TGFβ2 and IGF1 in AD-MSCs may produce chondrocytes that are slightly inferior to BM-MSCs chondrocytes, as measured using chondrocytes markers including COL1A, COL2A1, and SOX-9 (12).

The insulin-like growth factor (IGF1) is an enhancer that is responsible for the rate of gene expression (13–16), and IGF-2 plays a role as a growth stimulant in a non-differentiated state and as a regulator for glucose in all stages of differentiation (17, 18). A study reported that plasmid-based upregulation of IGF-1 in rabbit chondrocytes encapsulated using alginate *in vivo* showed an ability to repair cartilage and accelerated subchondral bone reformation in osteochondral disorders (19). Thus, we aimed to observe IGF-1 induction effect on the gene expression of chondrogenic markers, SOX9 and COL2, in Wharton’s Jelly MSCs.

## Materials and Methods

***Cell culture preparation***

The human Wharton’s Jelly mesenchymal stem cells (hWJMSCs) of passage 4 (P4) were collected from the Stem Cell and Cancer Institute (Jakarta, Indonesia). The cells had been characterized by the cell multipotent differentiation and surface phenotype (11, 20). Informed consent was obtained from the Institutional Ethics Committee at the Stem Cell and Cancer Institute (Jakarta, Indonesia).

The hWJMSCs at a density of 5 x 10^5^/well were cultured in minimum essential medium-α (α-MEM) (Gibco, 12561056), supplemented with fetal bovine serum (20%) (FBS, Gibco, 10270106) and 1% anti-biotic and anti-mycotic (Gibco, 15240062). They were incubated in a humidified atmosphere with 5% CO_2 _at 37 ^°^C for 24 hr. The medium was discarded and washed with Phosphate Buffered Saline (PBS).

hWJMSCs at density 1 x 10^6 cells/well was maintained in a complete medium. The cells were treated with IGF-1 (Biolegend, 590904) at concentrations of 75, 150, and 300 ng/ml, and incubated at 5% CO_2_, 37 ^°^C for 7 days, to obtain IGF1-induced WJMSCs cells for measuring SOX9 and COL2 gene expression (11, 20).

***Co-culture of IGF1-WJMSCs and IL1β-CHON002***

IGF1 75 ng/ml-induced WJMSCs, IGF1 120 ng/ml-induced WJMSCs (IGF1-WJMSCs), IL1β 5 ng/ml-induced CHON002, and IL1β 10 ng/ml-induced CHON002 (IL1β-CHON002) cells were collected with PBS containing 0.15% (w/v) trypsin (2000 units/g) and 0.02% EDTA. The cell suspensions of IGF1-WJMSCs and IL1β-CHON002 were mixed at five ratios including WJMSCs alone, IGF1-WJMSCs and IL1β-CHON002 1:2, 1:1, 2:1, IGF1-WJMSCs alone, IL1β-CHON002 alone, and CHON002 alone. The cells were stored at 37 ^°^C in 5% CO_2_, with medium changes three times per week. The co-culture was stained with alcian blue, after 2 weeks of incubation. Meanwhile, the conditioned medium (CM) was collected from the co-culture and evaluated by ELISA assay (21). 

***Quantification of COL2 and SOX9***

RNA was extracted using the Aurum total RNA kit (Bio-Rad, 7326820) and checked its concentration. The RNA was used for cDNA synthesis using iScript cDNA synthesis kit (Bio-Rad, 1708890) at 25 ^°^C temperature for 5 min, 42 ^°^C for 30 min, and 85 ^°^C for 5 min for the final step. The end-product was stored at -20 ^°^C.

Quantitative gene expression was conducted using Thermo Scientific PikoReal Real-time PCR System (Thermo Fisher). PCR followed these condition: pre-incubation cycle (95 ^°^C for 5 min), 40 cycles of denaturation (95 ^°^C for 1 min), annealing (47 ^°^C for 40 sec for SOX9, and 52 ^°^C for 40 sec for COL2), and extension (72 ^°^C for 1 min). The reaction mix that was used to perform qPCR was from an Evagreen master mix (Bio-Rad, 1725200). Table 1 shows the primers used in this research.

***Levels of ADAMTS1, ADAMTS5, MMP1, MMP3, and RANKL***

Levels of ADAMTS1, ADAMTS5, MMP1, MMP3, and RANKL were assessed in accordance with manufacturer’s protocols (Elabscience, H5539, H5590, H1441, H1446, and H2400, respectively). Briefly, 100 µl samples or standards were added into wells and incubated at 37 °C for 90 min before they were discarded. 100 µl of biotinylated was added, stored at 37 ^°^C for 60 min, and washed with 350 µl wash buffer three times. Afterwards, the 100 µl of HRP conjugate was added, then incubated at 37 ^°^C for 30 sec and washed with 350 µl wash buffer five times. 90 µl of substrate was added and incubated at 37 °C for 15 min until it turned blue. Then, 50 µl of stop solution was added until yellow changes appeared. Absorbance test was carried out at 450 nm.

## Results

***Levels of SOX9 and COL2***

**Figure 1:**
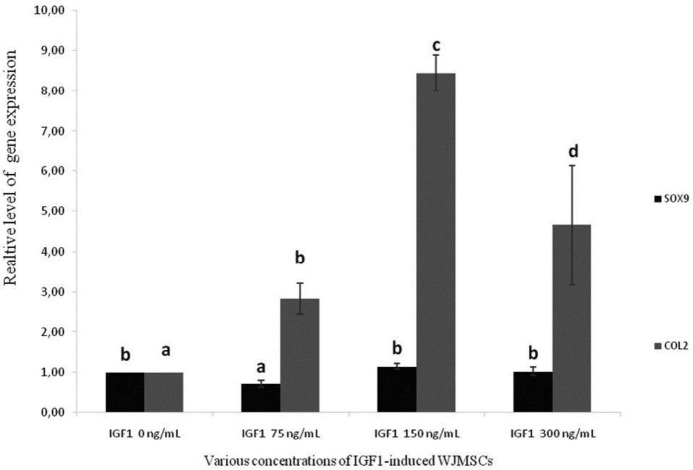
Gene expression levels of SOX9 and COL2 on IGF1-induced WJMSCs

SOX9 is present in differentiated chondrocytes and all chondrocyte progenitors during chondrogenesis. However, the expression is completely turned off in hypertrophic chondrocytes (22). SOX9 expression parallels that of the gene coding for COL2A1, a chondrocyte differentiation specific marker (23). SOX9 and COL2 were measured in WJMSCs induced by IGF1 (Figure 1).

Figure 1 shows that the expression of SOX9 and COL2 at IGF1 at concentration 150 ng/ml was higher, with values ​​of (1.15 ± 0.07) and (8.44 ± 0.44), respectively compared to IGF1 300 ng/ml, IGF1 75 ng/ml, and control. This indicates that IGF1-induced WJMSCs cells can improve chondrogenesis in controlling differentiation of chondrogenic cells, which can repair chondrocyte damage in OA. For co-culture of IL1β-induced CHON002 and IGF1-induced WJMSCs, we used IGF1 75 ng/ml and 120 ng/ml for inducing WJMSCs. 

**Table 1:** Primer sequences

Primer	Forward	Reverse
Beta actin	5’-TCTGGCACCACACCTTCTACAATG-3’	5’-AGCACAGCCTGGATAGCAACG-3’
SOX-9	5’-TTCGGTTATTTTTAGGATCATCTCG-3’	5’-CACACAGCTCACTCGACCTTG -3’
COL-2	5’-GGCAATAGCAGGTTCACGTACA -3’	5’-CGATAACAGTCTTGCCCCACTT -3’

*The data was presented as a histogram of mean±standard deviation. Different letters (a,b) are significant among various concentrations of IGF1 (0, 75, 150, and 300 ng/ml) toward level of SOX9 gene expression, different letters (a,b,c,d) are significant among concentrations of IGF toward COL2 gene expression based on Duncan’s *post hoc *test (*P*<0.05)

**Figure 2:**
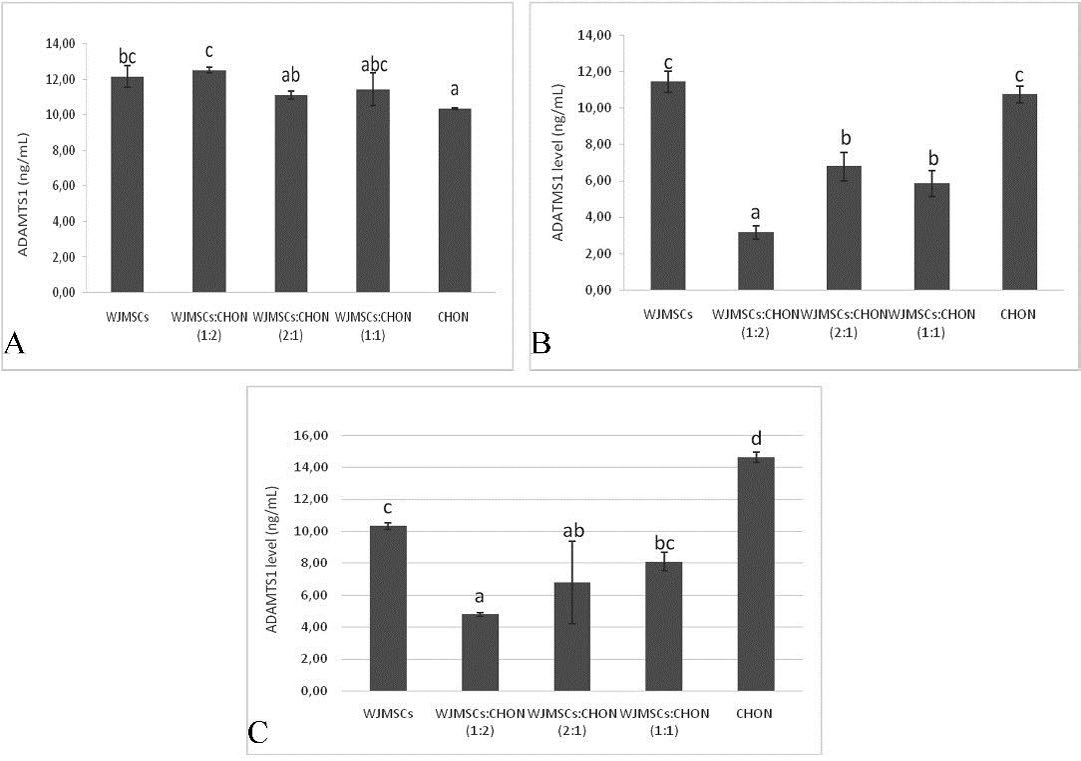
Levels of ADAMTS1 in IL1β-CHON002 and IGF1-WJMSCs co-cultures

A. Effect varied ratios of non-induced WJMSCs and non-induced CHON002 toward ADATMS1 level. Different letters (a,ab,abc,bc,c) are significant among ratios of WJMSCs and CHON002 (1:0, 1:2, 2:1, 1:1, 0:1) toward ADAMTS1 level based on Duncan’s *post hoc* test (*P*<0.05)

B. Effect varied ratios of IGF1 75 ng/ml-induced WJMSCs and IL1β 5 ng/ml-induced CHON002 toward ADATMS1 level. Different letters (a,b,c) are significant among ratios of IGF1 75 ng/ml induced-WJMSCs and IL1β 5 ng/ml-induced CHON002 (1:0, 1:2, 2:1, 1:1, 0:1) toward ADAMTS1 level based on Duncan’s *post hoc* test (*P*<0.05)

C. Effect varied ratios of IGF1 120 ng/ml-induced WJMSCs and IL1β 10 ng/ml-induced CHON002 toward ADATMS1 level. Different letters (a,b,c) are significant among ratios of IGF1 120 ng/ml induced-WJMSCs and IL1β 10 ng/ml-induced CHON002 (1:0, 1:2, 2:1, 1:1, 0:1) toward ADAMTS1 level based on Duncan’s *post hoc* test (*P*<0.05)

**Figure 3:**
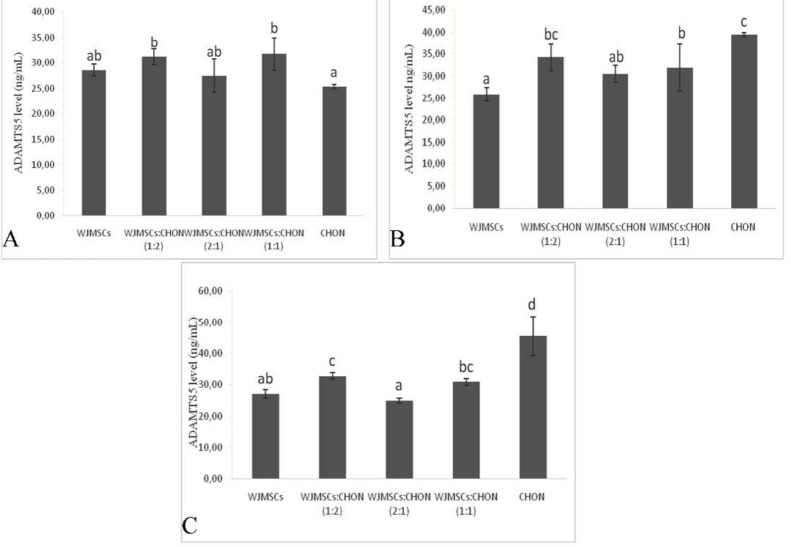
Levels of ADAMTS5 in IL1β-CHON002 and IGF1-WJMSCs co-cultures

A. Effect of varied ratios of non-induced WJMSCs and non-induced CHON002 toward ADATMS5 level. Different letters (a,ab,b) are significant among ratios of WJMSCs and CHON002 (1:0, 1:2, 2:1, 1:1, 0:1) toward ADAMTS5 level based on Duncan’s *post hoc* test (*P*<0.05)

B. Effect of varied ratios of IGF1 75 ng/ml-induced WJMSCs and IL1β 5 ng/ml-induced CHON002 toward ADATMS5 level. Different letters (a,ab,b,bc,c) are significant among ratios of IGF1 75 ng/ml induced-WJMSCs and IL1β 5 ng/ml-induced CHON002 (1:0, 1:2, 2:1, 1:1, 0:1) toward ADAMTS5 level based on Duncan’s *post hoc* test (*P*<0.05)

C. Effect varied ratios of IGF1 120 ng/ml-induced WJMSCs and IL1β 10 ng/ml-induced CHON002 toward ADATMS5 level. Different letters (a,ab,bc,c,d) are significant among ratios of IGF1 120 ng/ml induced-WJMSCs and IL1β 10 ng/ml-induced CHON002 (1:0, 1:2, 2:1, 1:1, 0:1) toward ADAMTS5 level based on Duncan’s *post hoc *test (*P*<0.05)

**Figure 4:**
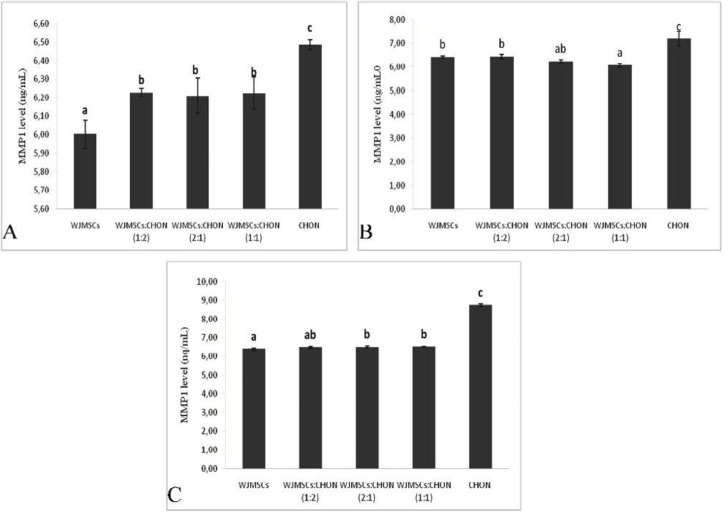
Levels of MMP1 in IL1β-CHON002 and IGF1-WJMSC co-cultures

A. Effect of varied ratios of non-induced WJMSCs and non-induced CHON002 toward MMP1 level. Different letters (a,b,c) are significant among ratios WJMSCs and CHON002 (1:0, 1:2, 2:1, 1:1, 0:1) toward MMP1 level based on Duncan’s *post hoc *test (*P*<0.05)

B. Effect of varied ratios of IGF1 75 ng/ml-induced WJMSCs and IL1β 5 ng/ml-induced CHON002 toward MMP1 level. Different letters (a,ab,b,c) are significant among ratios of IGF1 75 ng/ml induced-WJMSCs and IL1β 5 ng/ml-induced CHON002 (1:0, 1:2, 2:1, 1:1, 0:1) toward MMP1 level based on Duncan’s *post hoc *test (*P*<0.05)

C. Effect of varied ratios of IGF1 120 ng/ml-induced WJMSCs and IL1β 10 ng/ml-induced CHON002 toward MMP1 level. Different letters (a,ab,b,c) are significant among ratios of IGF1 120 ng/ml induced-WJMSCs and IL1β 10 ng/ml-induced CHON002 (1:0, 1:2, 2:1, 1:1, 0:1) toward MMP1 level based on Duncan’s *post hoc* test (*P*<0.05)

**Figure 5:**
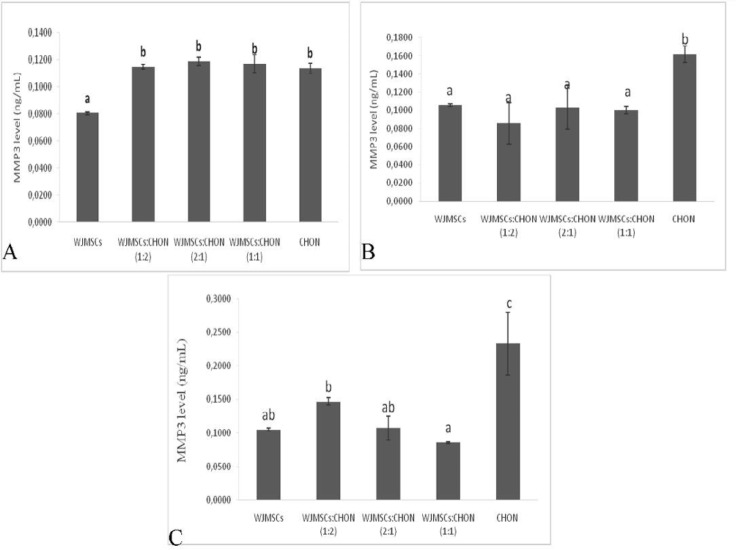
Levels of MMP3 in IL1β-CHON002 and IGF1-WJMSCs co-cultures

A. Effect of varied ratios of non-induced WJMSCs and non-induced CHON002 toward MMP3 level. Different letters (a,b) are significant among ratios of WJMSCs and CHON002 (1:0, 1:2, 2:1, 1:1, 0:1) toward MMP3 level based on Duncan’s *post hoc* test (*P*<0.05)

B. Effect varied ratios of IGF1 75 ng/ml-induced WJMSCs and IL1β 5 ng/ml-induced CHON002 toward MMP3 level. Different letters (a,b) are significant among ratios of IGF1 75 ng/ml induced-WJMSCs and IL1β 5 ng/ml-induced CHON002 (1:0, 1:2, 2:1, 1:1, 0:1) toward MMP3 level based on Duncan’s *post hoc *test (*P*<0.05)

C. Effect of varied ratios of IGF1 120 ng/ml-induced WJMSCs and IL1β 10 ng/ml-induced CHON002 toward MMP3 level. Different letters (a,ab,b,c) are significant among ratios of IGF1 120 ng/ml induced-WJMSCs and IL1β 10 ng/ml-induced CHON002 (1:0, 1:2, 2:1, 1:1, 0:1) toward MMP3 level based on Duncan’s *post hoc* test (*P*<0.05)

**Figure 6:**
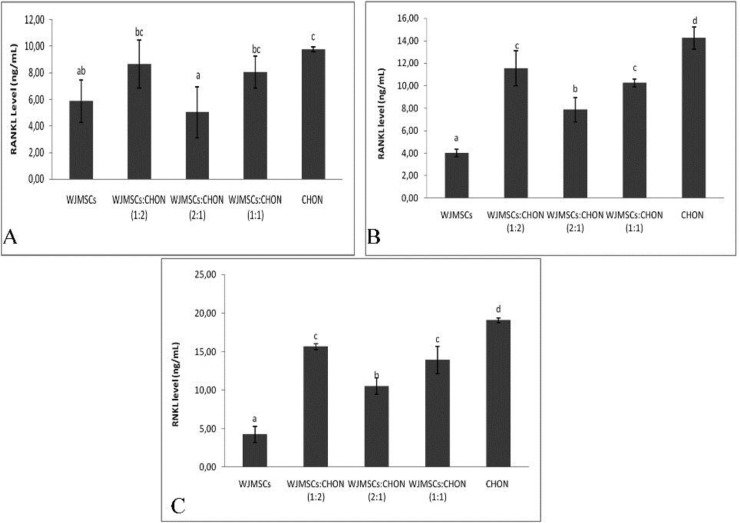
Level of RANKL in IL1β-CHON002 and IGF1-WJMSCs co-cultures

A. Effect of varied ratios of non-induced WJMSCs and non-induced CHON002 toward RANKL level. Different letters (a, ab, bc, c) are significant among ratios WJMSCs and CHON002 (1:0, 1:2, 2:1, 1:1, 0:1) toward RANKL level based on Duncan’s *post hoc* test (*P*<0.05)

B. Effect of varied ratios of IGF1 75 ng/ml-induced WJMSCs and IL1β 5 ng/ml-induced CHON002 toward RANKL level. Different letters (a,b,c,d) are significant among ratios of IGF1 75 ng/ml induced-WJMSCs and IL1β 5 ng/ml-induced CHON002 (1:0, 1:2, 2:1, 1:1, 0:1) toward RANKL level based on Duncan’s *post hoc* test (*P*<0.05)

C. Effect of varied ratios of IGF1 120 ng/ml-induced WJMSCs and IL1β 10 ng/ml-induced CHON002 toward RANKL level. Different letters (a,b,c,d) are significant among ratios of IGF1 120 ng/ml induced-WJMSCs and IL1β 10 ng/ml-induced CHON002 (1:0, 1:2, 2:1, 1:1, 0:1) toward RANKL level based on Duncan’s *post hoc* test (*P*<0.05)

***Level of ADAMTS1 and ADAMTS5***

ADAMTS1 is presented within cartilage and the synovium (24) and its expression is significantly upregulated in OA cartilage (25). ADAMTS1 and ADAMTS5 levels are shown in Figures 2 and 3, respectively.

In general, co-cultures of IGF1-induced WJMSCs and IL1β- induced CHON002 with ratio 1:2 showed lowest ADAMTS1 among treatments (Figure 2); co-culture of IGF 75 ng/ml-induced WJMSCs and IL1β 5 ng/ml-induced CHON002 (1:2) showed lowest ADAMTS1 level (3.15 ng/ml), and it was significantly different compared to the positive control, IL1β 5 ng/ml-induced CHON002, and IL1β 10 ng/ml-induced CHON002 (10.74 and 14.64 ng/ml, respectively). However, there was no marked difference between IGF1-induced WJMSCs alone, in which ADAMTS1 levels in both treatments (IGF1 75 ng/ml, 120 ng/ml) were 11.44 and 10.31 ng/ml, respectively.

On the other hand, co-culture IGF1 120 ng/ml-induced WJMSCs and IL1β 10 ng/ml-induced CHON002 (2:1) showed the lowest ADAMTS5 level (24.83 ng/ml), which was significantly different compared to positive control, IL1β 5 ng/ml-induced CHON002, and IL1β 10 ng/ml-induced CHON002 (39.48 and 45.58 ng/ml, respectively). The result was comparable to negative control, CHON002 alone without induction (25.36 ng/ml). Single culture and IGF1-induced WJMSCs alone (IGF1 75 ng/ml, 120 ng/ml) also presented the low levels of ADAMTS5 (25.89 and 26.95 ng/ml, respectively).

***Level of MMP1 and MMP3***

The levels of MMP1 and MMP3 in co-culture IGF1-WJMSCs and IL1β-CHON002 are shown in Figures 4 and 5, respectively.

The results of the present study show that both IGF1-WJMSCs itself and co-culture with IL1β-CHON002 significantly decreased the level of MMP1 (Figure 4) compared to positive control namely, IL1β 5 ng/ml-induced CHON002 and IL1β 10 ng/ml-induced CHON002 (7.19 and 8.73 ng/ml, respectively). These results were comparable to negative control, CHON002 alone without induction (6.49 ng/ml). Similar results were also found in MMP3 levels in which both IGF1-induced WJMSCs alone and co-culture significantly decreased the MMP3 level compared to IL1β-induced CHON002 (Figure 5).

***Level of RANKL***

The TNF molecules called RANKL (receptor activator of NFκB ligand) is the main regulator of bone remodeling and development and activation of osteoclasts. The levels of RANKL can be seen in Figure 6.

The results obtained show that there was a significant difference in RANKL levels between the treatment of IGF1-WJMSCs and IL1β-CHON002 (Figure 6). Level of RANKL was reduced by both IGF1-WJMSCs alone and co-culture, and it was significant compared to the negative control, IL1β 5 ng/ml-induced CHON002, and IL1β 10 ng/ml-induced CHON002. Co-culture IGF1 75 ng/ml-induced WJMSCs and IL1β 5 ng/ml-induced CHON002 (2:1) showed the lowest RANKL levels among co-cultures.

## Discussion

MSCs have been employed as one particular sector of tissue engineering that involves the repair, replacement, or regeneration of cartilage tissue, due to their superior proliferative and differentiation capacities (26). It has been reported that WJMSCs may differentiate into chondrocytes, skeletal muscle cells, cardiac muscle cells, osteoblasts, adipocytes, β cells in the islets of Langerhans, or endothelial cells *in vitro* (27). Hence, these cells may be applied in the treatment of chronic degenerative disorders and prevent cartilage degradation in patients with OA through their trophic/regenerative potential. 

In the present study, treating WJMSCs with IGF1 increased expression of SOX9 (Figure 1). Many cartilage matrix genes have been indicated to be under the regulation of transcriptional control of SOX9. They include COL2A1*, *COL9A1*, *COL11A2, aggrecan, and cartilage link protein (CRTL1) genes (28–31), all of which are involved in articular cartilage structure and function. Furthermore, SOX9 is presented and present in presumptive cartilage during embryo development. The mutations in human SOX9 gene leads to campomelic dysplasia with skeletal malformation and dwarfism (32). Thus, downregulation of SOX9 in OA is clearly likely to contribute to cartilage pathology. 

Moreover, induction of IGF1 150 ng/ml in WJMSCs also increased expression of COL2 (Figure 1). Referring to previous studies, SOX9 overexpression in human chondrocytes increases COL2A1 expression, as well as their capacity to reform a cartilage ECM (33–35). Collagenase-1, -2, and -3 are all synthesized by chondrocytes and have been considered as the rate-limiting enzymes in collagen degradation (36–38). Collagenase levels in the synovial fluid and serum correlate with cartilage destruction in OA (39). An imbalance between collagenases and their endogenous inhibitors has also been suggested to result in cartilage collagenolysis (40). 

The results obtained in this study are in line with previous studies that proteoglycan core protein and collagen type II are induced by IGF1 and that it stabilizes the chondrocyte phenotype in pathological conditions (41–43). IGF-1 is fairly mitogenic in human adult articular cartilage and highly stimulates the production of chondrocyte extracellular matrix components (13, 14).

Furthermore, IGF1 with concentrations of 75 ng/ml and 120 ng/ ml was used to induce WJMSCs which were co-cultured with IL1β (5 ng/ml, 10 ng/ml)-induced CHON002. These IGF1 concentrations were used in this study because SOX9 expression is not appropriate. According to Kim and Im, 2009, supplementation of mesenchymal stem cells with IGF, did not induce SOX9 significantly (44). Parameters measured were chondrogenic markers that included ADAMTS-5, ADAMTS-1, MMP-3, MMP-1, and RANKL. Elevation of MMP-1 (collagenase-1) and MMP-3 (stromelysin-1) have been documented in osteoarthritic cartilage (45, 46) and in the synovial fluid of osteoarthritic joints (47, 48). The present study has recognized the messenger RNA (mRNA) presence for some MMPs, i.e., MMP13, MMP9, MMP3, and MMP1, in human OA cartilage (49, 50), and other studies have reported specific MMP proteins and collagenase-mediated type II collagen degradation products (51, 52). These enzymes are involved in intrinsic chondrocyte-mediated degenerative changes of the cartilage matrix in OA.

In the present study, IGF-induced WJMSCs decreased the ADAMTS1 level. ADAMTS1 has shown in cartilage and the synovium (24) to cleave aggrecan and versican (53). Some studies reported that the expression of ADAMTS1 is significantly elevated in OA cartilage (25, 54–58), however, some studies also indicated a decreased expression in late-stage human OA (59–61).

The TNF family of molecules called RANKL (receptor activator of NFκB ligand, also known as osteoprotegerin ligand), osteoclast differentiation factor (ODF), TNF related activation-induced cytokine (TRANCE), and TNFSF11 and its receptor RANK (TNFRSF11A) is the main regulator of bone remodeling and activation of osteoclasts (62, 63–66). In this study, IGF-induced WJMSCs also reduced the RANKL level. Production of RANKL activated by T-cells directly controls osteoclastogenesis, bone remodeling, and also associated with autoimmune diseases, cancers, leukemias, asthma, chronic viral infections, and periodontal disease (63). In particular, RANKL is more likely to be the pathogenetic principle that results in the destruction of bone and cartilage in arthritis. RANKL is highly presented in osteoblast/stromal cells, primitive mesenchymal cells surrounding the cartilaginous anlagen, and hypertrophying chondrocytes (62). RANKL mRNA has also been observed in hypertrophic and prehypotrophic chondrocytes at day 15 of embryogenesis and extraskeletal tissues such as the brain, heart, kidneys, skeletal muscles, and skin throughout mouse development (67). RANKL expression can be upregulated by bone-resorbing factors i.e., vitamin D3, glucocorticoids, IL1, IL6, IL11, IL17, TNFα, PGE_2_, and PTH (68, 62, 65). 

These findings are supported by several studies. A study done by Ahmed *et al.* showed that both rat model and co-culture between MSCs and cartilage chips involve MMP-13 and tissue inhibitor of MMP1 and MMP2 as factors in hypertrophy (69).  Moreover, some studies also reported enhanced chondrogenesis of MSCs co-cultured with chondrocytes, which shows higher cartilage-specific marker expression in the co-culture compared with monocultures as well as reduced expression of hypertrophic markers such as MMP3 (70, 71). IGF1-WJMSCs is, therefore, promising for use as medicine in the treatment of OA.

## Conclusion

The IGF1-induced WJMSCs increased expression of COL2 and SOX9 compared to controls, which indicates IGF1-WJMSCs are capable of enhancing chondrogenesis and can be further used in OA treatment. Validation of IGF1-WJMSCs in animal models should eventually follow as further study. 
